# In Vitro Models for Studying Entry, Tissue Tropism, and Therapeutic Approaches of Highly Pathogenic Coronaviruses

**DOI:** 10.1155/2021/8856018

**Published:** 2021-06-21

**Authors:** Saeid Najafi Fard, Linda Petrone, Elisa Petruccioli, Tonino Alonzi, Giulia Matusali, Francesca Colavita, Concetta Castilletti, Maria Rosaria Capobianchi, Delia Goletti

**Affiliations:** ^1^Translational Research Unit, Epidemiology and Preclinical Research Department, National Institute for Infectious Diseases “Lazzaro Spallanzani” IRCCS, 00149 Rome, Italy; ^2^Laboratory of Virology, Epidemiology and Preclinical Research Department, National Institute for Infectious Diseases “Lazzaro Spallanzani” IRCCS, 00149 Rome, Italy

## Abstract

Coronaviruses (CoVs) are enveloped nonsegmented positive-sense RNA viruses belonging to the family Coronaviridae that contain the largest genome among RNA viruses. Their genome encodes 4 major structural proteins, and among them, the Spike (S) protein plays a crucial role in determining the viral tropism. It mediates viral attachment to the host cell, fusion to the membranes, and cell entry using cellular proteases as activators. Several in vitro models have been developed to study the CoVs entry, pathogenesis, and possible therapeutic approaches. This article is aimed at summarizing the current knowledge about the use of relevant methodologies and cell lines permissive for CoV life cycle studies. The synthesis of this information can be useful for setting up specific experimental procedures. We also discuss different strategies for inhibiting the binding of the S protein to the cell receptors and the fusion process which may offer opportunities for therapeutic intervention.

## 1. Introduction

Coronaviruses (CoVs) are enveloped nonsegmented positive-sense RNA viruses belonging to the family Coronaviridae, order Nidovirales, broadly distributed in humans, and other mammals [1]. They can cause varieties of diseases, including respiratory, enteric, renal, and neurological syndromes [2].

With 80–160 nm in diameter, CoVs contain the largest viral genome (27–32 kilobase pairs) among the RNA viruses, sharing similar genome organization [3, 4]. They encode for 14-16 nonstructural proteins and 4 structural proteins, the spike (S) glycoprotein, small envelope protein (E), integral membrane protein (M), and genome-associated nucleocapsid protein (N) [3, 5, 6]. In addition, genes encoding accessory proteins have also been identified in the three regions between S–E–M–N [3, 4]. The proteins E, M, and N are mainly responsible for virions assembly while the S protein mediates viral attachment, membrane fusion, and entry, thus, determining tissue and cell tropism as well as host range [5, 7].

CoVs are classified into four genera, including alpha-, beta-, gamma-, and delta-CoVs [8]. So far, there have been three zoonotic outbreaks of beta-CoVs. In 2002–2003, severe acute respiratory syndrome coronavirus (SARS-CoV), a lineage B beta-CoV (subgenus sarbecovirus), emerged and infected over 8,000 people causing about 800 deaths [8, 9].

In 2012, Middle East respiratory syndrome coronavirus (MERS-CoV), a lineage C beta-CoV (subgenus *Merbecovirus*), was discovered as the etiologic agent of a severe respiratory syndrome in Saudi Arabia, that caused 2,494 confirmed cases and 858 deaths [10]. Nowadays, sporadic cases are registered, remaining endemic in Middle East [11]. In December 2019, cases of pneumonia of unknown cause were reported in Wuhan, Hubei Province, China, that were later confirmed to be caused by a novel coronavirus, named SARS-CoV-2 due to its close phylogenetic relation to bat SARS-like CoVs. The clinical condition caused by this novel coronavirus is called COronaVIrus Disease-19 (COVID-19). SARS-CoV-2, a lineage B beta-CoV, is the third known highly pathogenic human coronavirus infection in the last two decades after SARS-CoV and MERS-CoV [2, 8, 12].

In addition to the highly pathogenic zoonotic pathogens, four coronaviruses with low-pathogenicity are endemic in humans. Among them, human CoV 229E (HCoV-229E) and HCoV-NL63 belong to alpha-CoVs, while HCoV-OC43 and HCoV-HKU1 are members of beta-CoVs [4, 13, 14].

**Figure 1:**
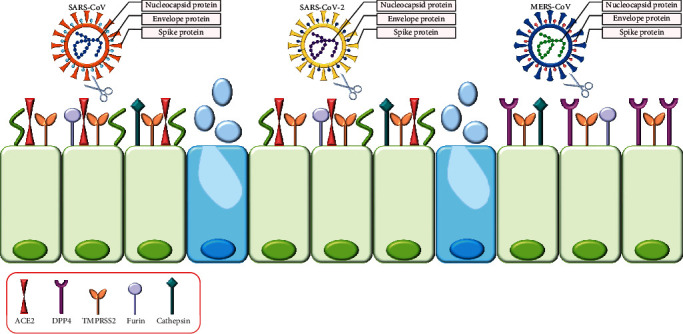
Coronaviruses (CoVs) are enveloped nonsegmented positive-sense RNA viruses belonging to the family Coronaviridae. Four genera are known: alpha-, beta-, gamma-, and delta-CoVs. Beta-CoVs are accountable for zoonotic outbreaks as severe acute respiratory syndromes- (SARS-) CoV, Middle East respiratory syndrome virus- (MERS-) CoV, and now, the novel SARS-like coronavirus (SARS-CoV-2). CoVs encode several nonstructural and structural proteins as genome-associated nucleocapsid protein (N), envelope protein (E), and spike (S) glycoprotein. In particular, S protein is crucial for viral attachment, fusion, and entry into epithelial cells. SARS-CoV and SARS-CoV-2 bind to ACE2 receptor highly expressed on ciliated epithelial cells, whereas MERS-CoV enters nonciliated epithelial cells binding the CD26/DPP4 receptor. Several host cellular proteases are needed to activate the S protein (e.g., TMPRSS2, furin, and cathepsins) and viral entry into cells.

Several in vitro models have been used to study the biology of pathogenic coronaviruses, so far. SARS-CoV-2 was first isolated using human airway epithelial cells [2]. SARS-CoV, SARS-CoV-2, and MERS-CoV can be isolated and grow readily in Vero cells [9, 12, 15–17]. The critical first step for CoVs infection is the entry into the susceptible host cells by binding to a specific receptor. SARS-CoV and SARS-CoV-2 recognize angiotensin-converting enzyme 2 (ACE2), whereas MERS-CoV recognizes dipeptidyl peptidase 4 (DPP4, also referred as cluster of differentiation 26, CD26) as its receptor (Figure 1) [18–20].

In addition to the requirement of receptors for cellular entry, several cellular proteases have been reported to be key factors for CoVs infection, which determine the entry route into host cells through cleavage of the S protein and activation of its membrane fusion activity [21–25].

Studies have shown that both human and animal cell lines can express ACE2 and CD26 receptors and can be used to study the mechanism of viral entry and host species restriction of different CoVs, although the expression levels and regulation of these receptors are not yet completely elucidated [8, 26–33]. Different receptors are used for cell entry, and several models have been applied for in vitro studies on CoVs; however, based on our current knowledge, a comprehensive review of in vitro studies and models on CoV pathogenesis is missing. For instance, data related to the expression levels of ACE2 and CD26/DPP4 in human and animal cell lines are highly controversial, probably due to the method of detection and reagents used. Here, we aim to review in vitro studies on highly pathogenic CoVs focusing on ACE2 and CD26/DPP4 receptor expression, cell tropism, and models useful for better understanding the viral life cycles, which could potentially offer opportunities for therapeutic intervention.

## 2. Methods

### 2.1. Search Strategy

Medline, EMBASE, Scopus, and Web of Science databases were used to perform the review. Studies were identified by crossing the name coronavirus or COVID-19 with the following key terms: SARS-CoV, MERS-CoV, SARS-CoV-2, Viral entry, ACE2 and/or CD26/DPP4 receptors, cellular proteases, and cell lines. We included data from English language articles. Meeting abstracts not yet published as full article, and case reports were excluded. The literature review was extended to April 15, 2021.

## 3. Results

### 3.1. Cellular Tropism of Highly Pathogenic Human Coronaviruses

The cell and tissue tropism of viruses is defined as the capacity of a virus to infect specific cells and tissues and is determined by both viral and cellular factors. Indeed, it depends on virus attachment protein(s) (antireceptors) and the presence of the cognate cellular receptor(s). The ability of CoVs to infect a wide range of mammals and birds is primarily determined by the ability of the S protein to bind a cell surface receptor [1]. Moreover, their zoonotic potential is due to the adaptability of S proteins to receptors of other species [18]. The receptor binding is the first critical step for the virus tropism. Although both SARS-CoV and SARS-CoV-2 employ ACE2 as a receptor for cellular entry [19], the SARS-CoV-2 S protein binds ACE2 with higher affinity than SARS-CoV S, which may explain the greater transmissibility of SARS-CoV-2 [20].

Of note, both SARS-CoV and SARS-CoV-2 have a marked tropism for ciliated cells widely distributed along the upper and lower respiratory tract [19, 29]. Key factors for CoVs entry into cells are host cellular proteases such as type II transmembrane serine protease (TMPRSS2), furin, and members of the cathepsin family (e.g., B and L) [21–23, 25, 34–36]. Indeed, the S protein is cleaved by cellular proteases in the priming step. As the priming event is essential for virus entry, the efficacy and extent of this activation step should regulate cellular tropism and viral pathogenesis of the different CoVs [37].

### 3.2. Structure and Function of Coronavirus S Proteins

The S proteins are a class I viral glycoproteins, which form homotrimers protruding from the viral surface that undergo structural modifications during host receptor binding and virus-host cell membrane fusion steps. They are divided into an N-terminal S1-ectodomain that recognizes a cognate cell surface receptor and a C-terminal S2-membrane-anchored protein responsible for virus–host membrane fusion and viral entry [4, 37–40].

The S1 region contains two major domains, the N-terminal domain (NTD) and C-terminal domain (CTD) that can both function as receptor-binding domain (RBD). RBDs are divided into a core subdomain and a receptor-binding motif (RBM). Among CoVs, the core subdomain is structurally similar while the RBMs differ significantly, thus, explaining the peculiar receptor binding. Upon S1 binding to the host receptor, a conformational change in S2 mediates fusion between the viral envelope and the cell membrane. The viral genome is then released into the cytoplasm of the host cell through the fusion core [4, 14].

#### 3.2.1. The SARS-CoV S Protein

The S of SARS-CoV is a homotrimer that belongs to a group of class I viral fusion glycoproteins that includes HIV glycoprotein 160 (Env), influenza haemagglutinin (HA), paramyxovirus F, and Ebola virus Gp2 [41]. The SARS-CoV S gene encodes a surface glycoprotein precursor of 1,255 amino acids in length. The amino terminus and most of the protein are predicted to be on the outside of the virus particle surface [42]. The S protein can be cleaved into the S1 and S2 subunits by proteases, and the fusion process is similar to that mediated by class I viral fusion proteins of other viruses (e.g., HIV-1 and murine hepatitis virus (MHV)), but may occur in the acidic environment of the endosomes, rather than on the cell surface [43, 44]. S2 contains heptad repeat 1 (HR1) and HR2 domains, which play an important part in virion fusion with target cells [45]. The S protein of SARS-CoV binds to its cellular receptor, ACE2. A defined RBD on S mediates this interaction. The atomic details at the interface between the two proteins clarify the importance of residue changes that facilitate efficient cross-species infection and human-to-human transmission [40].

#### 3.2.2. The MERS-CoV S Protein

The S protein of MERS-CoV, similarly to SARS-CoV, is a type I transmembrane glycoprotein in a trimer state, which is located at the viral envelope surface. It contains 1,353 amino acids that can be cleaved into a receptor-binding subunit S1 and a membrane-fusion subunit S2 [29, 46]. S1 is located in the N-terminal 14–751 amino acids, containing the RBD region, which binds CD26/DPP4 [47, 48]. Although the RBDs of MERS-CoV and SARS-CoV share a high degree of structural similarity in their core subdomains, their RBMs are quite different, which can explain the different receptors utilized by these viruses [49, 50]. After binding to cellular receptor, MERS-CoV S protein undergoes significant conformational changes, exposing HR1 and HR2 regions in S2 subunit, further forming a 6-helix bundle fusion core, which leads to membrane fusion [51, 52].

#### 3.2.3. The SARS-CoV-2 S Protein

The S protein of SARS-CoV-2 shares about 76% of amino acid identities with SARS-CoV, and, in particular, the sequence of potential RBD of SARS-CoV-2 is only about 74% homologous to that of SARS CoV [53]. Studies have reported that SARS-CoV-2 uses ACE2 as the cellular receptor for entry [8]. Successful conformational changes of S protein that lead to membrane fusion not only require receptor binding but also need appropriate protease activation. The cell-surface serine protease TMPRSS2 makes S protein fusogenic [54]. Moreover, there is a furin site between S1 and S2 (amino acids 682-685, motif of Arg-Arg-Ala-Arg (RRAR)) subunits in SARS-CoV-2 S protein, but it is not clear whether this furin site has any effect on viral pathogenesis and virus spreading among humans [8, 55].

## 4. Viral Attachment and Entry

### 4.1. Receptors for Highly Pathogenic Coronaviruses

Several types of receptors have been identified for alpha and beta-CoVs to enter cells [40]. hCoV-229E uses Aminopeptidase N (APN, CD13) as its receptor and OC43 and HKU1 attach to O-acetyl-sialosides found on glycoproteins and glycolipids at the host cell surface to enable entry into susceptible cells [4, 14, 38]. As mentioned in previous sections, ACE2 has been identified as the specific receptor for SARS-CoV and SARS-CoV-2 [20], while MERS-CoV utilizes CD26/DPP4 as its cellular receptor [49].

ACE2, a type I membrane protein, has a single metalloprotease active site and a transmembrane domain. It cleavages angiotensin (Ang) I to produce Ang-(1-9) and provides a direct binding site for the S proteins of some CoVs [56]. Study of the expression of ACE2 protein, the tissue tropism, and cellular distributions of SARS-CoV and SARS-CoV-2 is crucial to understand the mechanisms of pathogenesis of these viruses. ACE2 is widely expressed in different body sites including lung, heart, kidney, testis, intestine epithelia, arterial and venous endothelial cells, and maternal-fetal interface [19, 57, 58]. In the respiratory tract, ACE2 is expressed on the epithelial cells of alveoli, trachea, and bronchi, bronchial serous glands, and alveolar monocytes and macrophages [59]. ACE2 without ectopeptidase activity is also an efficient SARS-CoV receptor, and several proteases can operate as SARS-CoV entry cofactors, including cathepsin L, elastase, trypsin, factor Xa, thermolysin, and plasmin [23]. Moreover, a truncated form of the cytoplasmic domain of ACE2 does not affect the efficiency of SARS CoV infection in a pseudotype assay, suggesting that this domain is not essential for ACE2-mediated entry of the virus [60].

Previous studies have shown that certain ACE2-expressing endothelial cells and human intestinal cell lines failed to be infected by SARS-CoV [61, 62]. The SARS-CoV cannot replicate in differentiated respiratory epithelial cells or in primary undifferentiated bronchial and alveolar epithelial cells, even when ACE2 is abundantly expressed [63]. On the other hand, type II pneumocytes and bronchial epithelial cells differentiated in vitro could be infected with SARS-CoV [64]. In contrast, some cells without a detectable expression level of ACE2 such as hepatocytes could be infected by SARS-CoV, suggesting that ACE2 presence alone is not sufficient for sustaining viral infection [65]. Altogether, these observations indicate that different receptors or coreceptors may be utilized by SARS-CoV for infecting different tissues.

Indeed, SARS-CoV has been reported to also interact with alternative receptors, such as dendritic cell-specific intercellular adhesion molecule-3-grabbing non-integrin as (DC-SIGN) and liver/lymph node SIGN (L-SIGN, e.g., CD209L) [66–68]. DC-SIGNR is expressed in alveolar type II cells, the major cell type infected in fatal cases of SARS infection [69]. However, these proteins appear to be much less efficient than ACE2 in promoting SARS-CoV entry and do not support infection effectively in absence of ACE2 [45, 70].

Vimentin, an intermediate filament, has been identified as abundantly present in the SARS-CoV S protein-ACE2 complexes Vimentin may directly bind to the SARS-CoV S protein, suggesting that it may also serve as a coreceptor for SARS-CoV [68]. Interestingly, it has been preliminary reported that antibodies directed against Vimentin are able to prevent SARS-CoV-2 pseudovirus infection in vitro [71].

Regarding SARS-CoV-2, two studies published in Science identified the protein Neuropilin-1 (NRP1) as an additional cellular factor which may facilitate viral entry and infectivity [72, 73]. Moreover, a number of alternative SARS-CoV-2 entry factors, which may function as coreceptors for viral attachment, have been investigated including heparan-sulfates, Sialic Acid, L-SIGN/DC-SIGN CD147, glucose-regulated protein 78 (GRP78), angiotensin II receptor type 2 (AGTR2), and the receptor for advanced glycation end products (RAGE) [74].

Unlike SARS-CoV and SARS-CoV-2, MERS-CoV utilizes CD26/DPP4 as its main cellular receptor [49]. CD26/DPP4 is a 110 kDa type II transmembrane glycoprotein homodimer present on the cell surface, which can regulate hormone and chemokine activity by cleaving dipeptides at the N-terminus after a proline or alanine residue in the penultimate position [75]. The use of peptidases by CoVs may be related to their abundant presence on epithelial and endothelial tissues, the primary tissues of CoV infection [29, 48, 76]. CD26/DPP4 is also widely expressed on epithelial cells in kidney, small intestine, liver and prostate, and on activated leukocytes. In the human respiratory tract, it is mainly expressed on nonciliated epithelial in alveoli rather than the nasal cavity or conducting airways [29, 77]. Since CD26/DPP4 is a costimulatory factor for T cell activation, it is tempting to speculate a viral-mediated mechanism of the host immune system evasion [26, 59]. CD26/DPP4 is conserved among different mammals (e.g., bats, dromedaries, and humans), and the broad species tropism of MERS-CoV may partly be the result of this conservation [28, 78]. It has been also found that the susceptibility or resistance to the infection of different cell lines directly correlates with the presence or absence of CD26/DPP4 expression [27]. It has been shown that MERS-CoV pseudoviruses had a relatively higher infectivity in certain cells (e.g., in HT-1080, Hep-2, MT-2, and NBL-7) with undetectable CD26/DPP4 expression [79]. Moreover, HEK cells with undetectable CD26/DPP4 had relatively high MERS-CoV infectivity [80]. On the other hand, MERS-CoV pseudovirus showed a lower infectivity in CD26/DPP4 expressing A549 and 293T cells [81]. Altogether, these findings suggest that the infectivity of MERS-CoV might not be completely associated with the expression of CD26/DPP4. Thus, similar to SARS-CoV, MERS-CoV might also use alternative receptors that have not been identified yet.

Recently, Vankadari and Wilce modelled a homo-trimer structure of SARS-COV-2 S protein. They predicted that the S1 domain of SARS-COV-2 S protein potentially interacts with the human CD26/DPP4 and suggested that SARS-COV-2 may share infection modes with that of SARS-CoV and MERS-CoV [82]. However, the potential role of CD26/DPP4 in the pathogenesis of new emerged SARS-CoV-2 is yet to be delineated.

CoVs in addition to the protein-based receptors (i.e., ACE2 and CD26/DPP4) [20, 49] use low-affinity interactions with carbohydrates such as sialylated glycans or sialic acids for cell attachment [14, 38]. The N-terminal S1 subunit comprises four *β*-rich domains, designated A, B, C, and D, with domain A or B acting as receptor-binding domains in different coronaviruses. While the protein receptors are generally bound via S1 domain B, the sialoglycan-based receptors are mediated by the S1 domain A of S protein [38, 83]. For instance, MERS-CoV S protein selectively binds to sialic acid (Sia), and glycan array analysis revealed a preference for *α*2,3-linked Sia over *α*2,6-linked Sia. Interestingly, the *α*2,3-linked Sias are predominantly distributed in the upper and lower respiratory tracts of camels and humans, sites of MERS-CoV replication, respectively [83]. Furthermore, although HCoV-OC43 and HCoV-HKU1 receptors have not been fully identified, they bind the 9-O-acetylated-sialic acid modifications on glycoprotein-based receptors [13, 14].

Seyran and colleagues hypothesised that the sialic acid-binding domain of S protein allows a viral “surfing” on the epithelium for receptor scanning by SARS-CoV-2 [84]. However, it has been recently shown that the Sia cell surface depletion by neuraminidase treatment increased cell attachment by both SARS-CoV and SARS-CoV-2, because Sias present on ACE2 receptors do not allow a perfect binding between spike and ACE2 [85]. On the contrary, the binding of MERS-CoV was hampered by either Sia modifications, such as 5-N-glycolylation and (7,) 9-O-acetylation, or Sia cell surface depletion by neuraminidase treatment of Calu-3 cells [83, 85], thus, revealing a different role for Sias during CoV infections. In fact, Chu and colleagues showed that, likely, SARS-CoV-2 utilizes heparan sulfate as an attachment factor for both Calu-3 cells and lung explant infection [85].

The fundamental role of the glycosylation in the interaction between SARS-CoV-2 S proteins and ACE2 receptor has been recently investigated using glycomic-informed glycoproteomics and molecular dynamic simulations. Zhao and colleagues identified several glycosylated residues in both S proteins (i.e., N0074, N0149, N0234, and N0801) and ACE2 receptors (i.e., N053, N090, N103, N322, and N432), thus, providing a valuable tool to study the receptor binding step of the viral life cycle [86]. Work by others confirmed the complexicity and the importance of glycosylation in both S proteins [87] and ACE2 receptor [88]. Moreover, these authors modelled a possible role for glycans in antigenic shielding, highlighting the importance of glycosylation in the strategy of immunogen design for vaccination and of therapeutic strategies for viral inhibition [86].

#### 4.1.1. In Vitro Evaluation of Receptor Expression

Identification of relevant human cell line models of CoVs infection facilitates further laboratory investigations to have a better understanding of the viral entry, pathogenesis, and various therapeutic approaches. Studies have shown that both human and nonhuman cell lines can express CD26 and ACE2 and can be used to study the mechanism of viral entry, pathogenesis, and host species restriction of different CoVs; however, the expression levels of these receptors need to be well elucidated [8, 26–33]. Several studies have evaluated the expression of ACE2 and CD26/DPP4 in human and animal tissues and cell lines; however, data on the expression levels of these receptors are highly discrepant (Table 1). For instance, Hamming and colleagues reported high levels of ACE2 expression in A549 cells [58], but others observed low levels of it [70, 89] and found that exogenous overexpression of ACE2 was required to render A549 cells permissive to viral infection and suggested that this difference could be due to different lineages of cell lines that may have evolved during the in vitro cell culture.

Transfection of human ACE2 or CD26/DPP4 in nonsusceptible cells of different lineages or species permitted infection with SARS-CoV, SARS-CoV-2, or MERS-CoV, respectively (Table 2).

Some human (e.g., 293T, HEK293T, and HeLa) and nonhuman (e.g., COS-7, BHK) cell lines expressing hACE2 or hCD26/DPP4 are commonly used for experimentation because of their high transfectability. For example, hACE-2 expression in 293T cells resulted in the SARS-CoV and SARS-CoV-2 S pseudovirion binding and entry [8]. Also, expression of human and bat CD26/DPP4 in COS-7 cells allowed MERS-CoV S1–Fc cell surface binding and entry [29].

Nevertheless, the receptor alone may not be sufficient to explain the infectivity of CoVs in different cells, and other cellular factors may play a crucial role in completing the full life cycle of CoVs. In this regard, Chan and coauthors examined seven established human intestinal cell lines, DLD-1, HCT-116, HT-29, LoVo, LS-180, SW-480, and SW-620, for their susceptibility to SARS-CoV infection and found that only LoVo cells, which express intermediate levels of ACE2, were permissive. On the other hand, ACE2 was also found to be highly expressed in other cell lines that were not permissive to SARS-CoV infection [61]. The host factors that determine the susceptibility of the different cell types to CoVs infection are yet to be fully identified and, therefore, need of further investigations.

In studies of the expression levels of CoVs receptors, the method of detection and types of the antibodies (Abs) used are particularly important for receptor detection. For instance, while some studies have reported high levels of ACE2 expression in Calu-3, Caco-2, and Huh-7 cell lines by using different polyclonal Abs [8, 104], Liao and colleagues, by using a monoclonal anti-ACE2 Ab and total cell lysates, found that Calu-3 had elevated ACE2 levels compared to Caco-2 cells, while ACE2 was not detected in Huh-7. Moreover, by flow cytometry, ACE2 was detected on Calu-3 cells but not on Caco-2 or Huh-7 cells [106]. Using indirect immunofluorescence, in another study, Ren and coauthors found a higher number of Caco-2 cells with surface expression of ACE2 compared with Calu-3 cells. In addition, a faint band was visible when Calu-3 cells were analyzed by western blot [96].

ACE2 expression level in the CNS, and particularly in neurons, has not been well defined yet. Previous reports indicated very little expression of ACE2 RNA in the CNS [109] and in brain organoids [110, 111]. Accordingly, Yang and colleagues detected ACE2 protein expression at relatively low levels in human pluripotent stem cells- (hPSC-) derived cortical neurons by immunostaining [112]. However, others found widespread expression of ACE2 protein in both neurons and cells close to the lumen of the organoids [113, 114]. Furthermore, the enzymatic activity of ACE2 in human brain tissues and cerebrospinal fluid (CSF) [115, 116] has been indicated the significant level of ACE2 protein expression within the human CNS, suggesting that the mRNA level of ACE2 is not the best surrogate for ACE2 protein expression [113].

Specific models of neural cell lines have not been yet developed for the study of highly pathogenic coronaviruses [109]. Although a study showed that neural cell lines such as human oligodendroglioma-derived (OL) and rat (C6) origins can be susceptible to SARS-CoV infection, low levels of viral replication were observed compared to other susceptible cell lines such as Vero E6 or Caco-2 [117]. Recently, neural progenitor cells (NPCs), neurons, and microglia derived from human induced pluripotent stem cells (hIPSCs) have been used in in vitro, showing the potential of SARS-CoV-2 to infect CNS cells [110, 112, 113].

Taken together, using different lineages of cell lines and different methods may lead to different results; however, we should consider that while cell lines represent easy and useful tools for in vitro investigation on viral tropism and pathogenesis, these models have certain limitations, due to their inability to recapitulate complex and dynamic responses or cell-cell interactions happening in the whole organism.

Alternatively, primary culture systems such as human primary bronchiolar epithelial cells (HBEpC), human airway epithelium (HAE) culture models, human-derived organoids, or pseudostratified epithelia appear to be promising models to study the viral entry and pathogenesis of CoVs [29, 109, 118]. For example, the HAE culture model expressing relevant receptors for CoVs can recapitulate not only the morphological features of the human upper airway, which allows the visualization and the cultivation of several viruses, such as the newly emerging viruses like SARS-CoV-2, but also provide an excellent model for studying different steps of the viral life cycle and responses of the lung microenvironment to viral infections [118]. More recently, organoids that are miniaturized, simplified 3D structures of an organ (e.g., lung, kidney, intestine, liver, blood vessels, and brain) produced in vitro, offer another alternative to study tropisms and complex physiological or pathological processes of highly pathogenic coronaviruses more similarly to the in vivo condition (Table 2). They can be developed from either pluripotent stem cells or organ-specific progenitors through a self-organization process and provide very useful systems to study CoV life cycle as well as to test new antiviral compounds [109]. In particular, for the new SARS-CoV-2, several antiviral compounds have been tested such as interferon lambda, remdesivir, inhibitors of tyrosine kinases (Imatinib), inosine monphosphate dehydrogenase (the immunosuppressant mycophenolic acid, MPA), TMPRSS2 (camostat mesylate), or JAK kinases (baricitinib) [119–123].

### 4.2. The Role of Cellular Proteases in Viral Entry

Although the primary receptors of highly pathogenic CoVs, ACE2, and CD26/DPP4 are expressed on the host cells of almost all organs, these viruses mainly cause lung diseases. Furthermore, the distribution of the receptors does not strictly correlate with viral cell tropism suggesting that other factors are required for viral entry and replication into target cells [22, 58, 105].

Previous studies have shown that after receptor binding, the key factors for CoVs entry into cells are host cellular proteases such as trypsin, furin, cathepsins (a diverse group of pH-dependent endosomal and lysosomal proteases), elastase, hypoxanthine-aminopterin-thymidine (HAT), and TMPRSS2, as activators of the viral S protein, which is a prerequisite for the fusion of viral and host cell membranes during viral entry [15, 21–23, 25, 34, 35, 81, 91, 105, 124, 129, 132, 148].

As the priming event is essential for virus entry, the efficacy and extent of this activation step by the proteases of the target cells should regulate cellular tropism and viral pathogenesis [5, 37]. SARS-CoV infectivity was enhanced in culture cells by the addition of exogenous elastase, which enabled virus entry via the cell surface [128]. In addition, furin and cathepsins were showed to play a critical role in CoV spread and cytopathicity [15, 21, 91, 124, 148]. HAT could activate S protein for cell-cell fusion in the context of surrogate systems while it has been found coexpressed with the viral receptor in bronchial epithelial cells and pneumocytes in vivo [145]. Moreover, TMPRSS2 can cleave and activate S protein on the cell surface following receptor binding for cell-cell and virus-cell fusion, thereby allowing cathepsin-independent host cell entry [105, 131, 141, 145].

Highly pathogenic CoVs are able to use two cellular proteolytic pathways, either an endosomal or a nonendosomal pathway, to ensure the adequate processing of the S protein. In the absence of proteases at the cell surface, they can enter cells by an endosomal pathway, and the S protein is activated by cathepsin B/L in the endosome [141, 145, 149]. In contrast, in the presence of proteases at the cell surface such as trypsin, elastase, TMPRSS2, and HAT, which induce envelope-plasma membrane fusion, viral S proteins attached to the receptor are activated and the virus directly enters into the cell from the surface [23, 25, 105, 132, 141]. However, S protein primed by TMPRSS2, but not cathepsin B/L, appears essential for viral entry [22, 25, 105], while viral replication in the presence of proteases has been shown to be 100 times higher than via the endosomal pathway in Vero E6 cells [128, 150].

The role of host cell proteases in CoVs infection appears not limited to cleavage of the S protein. It has been suggested that ACE2 is also processed by host cell proteases playing an important role in SARS-CoV entry and pathogenesis [141, 151]. SARS-CoV S protein binding to ACE2 triggers ACE2 processing by a disintegrin and metallopeptidase domain 17 (ADAM17)/tumor necrosis factor-converting enzyme (TACE), which promotes the uptake of SARS-CoV by Vero E6 cells [141, 152, 153]. In addition, ACE2 may also be processed by TMPRSS2 and HAT, suggesting that ACE2 cleavage increases S protein-mediated entry [23]. However, the mechanism underlying augmentation of infection and the role of ACE2 proteolysis in protease-dependent S protein activation have not been well defined yet [151].

The distribution of proteases appears to be correlated with CoV infection in the lung, and results obtained with surrogate cell culture systems suggest that proteases may play a significant role in CoV spread in the human respiratory tract [22, 105, 141, 154]. Indeed, Matsuyama and colleagues showed that the localization of TMPRSS2-expressing cells in normal lung tissues, rather than ACE2-expressing cells, correlated to SARS-CoV infection in mild lesions, suggesting that TMPRSS2 may determine viral tropism at an early stage of SARS-CoV infection [22]. Altogether, these studies suggest that the higher infectivity of pathogenic CoVs in the lungs could be due to an enhancement of direct viral cell entry mediated by a diverse range of cellular proteases [22, 25, 81, 91, 105, 131].

## 5. In Vitro Models of Targeting Viral Entry as Therapeutics

The cell entry process of highly pathogenic CoVs is critical for pathogenesis of these viruses. There are four main strategies developed to inhibit the binding of the S protein to the cellular receptors and the fusion process: (i) using antireceptors antibodies, (ii) using soluble receptors, (iii) targeting of S protein via anti-S antibodies (anti S1 or S2), and (iv) targeting the cellular proteases involved in the priming of the S protein. All these strategies have been evaluated for the development of therapeutics against highly pathogenic CoVs.

### 5.1. Targeting the Cellular Receptor and the S Protein

The development of inhibitors targeting the interaction between the S1 domain, particularly RBD, and its receptor, or manipulation of the receptor levels may offer several opportunities for therapeutic intervention, as well as for vaccine-induced neutralizing antibodies. Previous studies have shown that several antibodies, peptides, or small compounds that bind ACE2 may be useful for the treatment of SARS-CoV or SARS-CoV-2 infections, either by blocking the S-protein-binding site or by inducing an ACE2 conformational change unfavorable for viral binding or membranes fusion [31, 155]. Anti-ACE2 antibodies or soluble forms of ACE2 blocked virus entry and replication in Vero E6 cells, thus, confirming that ACE2 is a functional receptor for SARS-CoV [45, 97].

In particular, a soluble form of ACE2 was shown to block the association of the S1 domain to the cells and an anti-ACE2 antibody inhibited SARS-CoV replication with an EC50 of 1.5 mg/mL [31]. Moreover, the catalytically inactive form of ACE2 conjugated to the Fc domain of human IgG1 potently inhibited SARS-CoV infection in Vero cells, with an EC50 of 2 nM [156]. In addition, smaller portions of the ACE2 ectodomain exhibited inhibitory activity. The soluble ectodomain of ACE2 was shown to inhibit S-bearing pseudotype entry [60].

Several studies indicate that CD26/DPP4-targeting therapeutic agents, including anti-CD26/DPP4 antibodies and CD26/DPP4 antagonist, may prevent the interaction between MERS-CoV RBD and CD26/DPP4, inhibiting MERS-CoV infection [49]. Indeed, anti-CD26/DPP4 polyclonal antibodies inhibited in vitro MERS-CoV infection of primary human bronchial epithelial cells and Huh-7 cells [29]. Anti-CD26/DPP4 monoclonal antibodies (2F9, 1F7, and YS110) blocked the interaction between the S protein and CD26/DPP4, thereby neutralizing MERS-CoV infectivity in Huh-7 and JKT/CD26 (human T cell leukemia expressing human DPP4) cells [78]. Human anti-CD26/DPP4 antibodies inhibited infection of susceptible bat (RoNi/7.1) cells and Huh-7 cells, in a dose-dependent manner [27].

As mentioned above, the S protein RBD is a major target for anti-CoV therapeutics, and most of neutralizing antibodies target this domain. Notably, RBD-specific mAbs have more potent neutralizing activity than those targeting the S1 region outside RBD or the S2 region, suggesting that CoV RBD could serve as a main target for developing antibody-based therapeutics [5, 49]. The RBD binding antibodies inhibited the association of S1 to ACE2 and neutralized the infection of SARS-CoV of Vero E6 cells [126]. Moreover, Mou and colleagues demonstrated that polyclonal Abs directed against the RBD region efficiently neutralize MERS-CoV infection in Huh-7 cells [136].

Different assay systems have shown that neutralizing antibodies can block binding of S1, which contains RBD, to its receptor and fully protect cells from CoV infection [126], demonstrating that the S is a target for neutralizing antibodies and that such antibodies are generated in SARS-CoV-infected patients [135, 140, 142].

Recent studies have demonstrated that SARS-CoV RBD specific monoclonal and polyclonal antibodies may cross-react with SARS-CoV-2 RBD protein and inhibit SARS-CoV-2 entry into hACE2-expressing 293T cells [39, 131]. In addition, SARS-CoV RBD-specific antibodies could cross-neutralize SARS-CoV-2 pseudovirus infection, suggesting the potential to develop SARS-CoV RBD-based vaccine for prevention of infection by SARS-CoV-2 and SARS-CoV [18, 39, 69, 131].

Recently, other attempts have been made to block the SARS-CoV-2 cell entry. For instance, S pseudovirions into 293/hACE2 cells were significantly prevented by preincubation of soluble hACE2 at both 10 *μ*g/ml and 50 *μ*g/ml [8]. Case and colleagues used either human mAbs or hACE2-Fc Receptor Decoy Proteins to inhibit SARS-CoV-2 infections on Vero E6 cells [157]. Importantly, soluble hACE2 is able to inhibit viral infection of engineered human blood vessel or kidney organoids [158], further supporting the notion that hACE2 is the receptor and soluble hACE2 might be used as a therapeutic inhibitor against SARS-CoV-2 infection. Recently, it has been proposed to use a chimeric bispecific molecule between soluble hACE2 and anti-CD16 in order to better recruit innate immune cells against the particles of the virus or infected cells [159].

A different approach to treat CoVs infections that appears to be an attractive alternative to traditional mAbs are the nanobodies (Nbs), the single-heavy-chain antibody variant also termed the VHH domain. Zhao and coauthors showed that Nbs significantly block RBD of MERS-CoV binding to CD26/DPP4 in HuH-7 cells [137]. Different Nbs have been recently shown to block either the SARS-CoV-2 S protein attachment to ACE2 in vitro [160] or efficiently neutralize SARS-CoV-2 infection [161, 162]. Since Nbs are small, stable, efficacious, and straightforward to produce to target the RBD of S protein, they can represent a great promise as potential therapeutics candidates.

S2 subunit of CoVs S-protein also appears to be an intriguing target for developing CoV fusion inhibitors [43, 46, 163, 164]. Liu and colleagues evaluated peptides derived from the membrane-proximal (HR2) and membrane-distal (HR1) heptad repeat region of the S protein as inhibitors of SARS-CoV infection of Vero cells, founding that HR2 peptides, but not HR1, were inhibitory [43]. Moreover, Lu and coworkers designed several peptides spanning the HR1 and HR2 sequences of MERS-CoV S protein and found that one of the HR2 peptides (HR2P) potently inhibited MERS-CoV infection and its S protein-mediated cell–cell fusion in Vero and Huh-7 cells, suggesting that these peptides can be further developed as effective MERS therapeutics [46].

Interestingly, a similar membrane fusion assay has been recently utilized to identify drugs with anti-SARS-CoV-2 activity. Braga and colleagues performed a microscopy-based screening with over 3000 approved drugs to search for inhibitors of SARS-CoV-2 S protein-driven syncytia [165]. Among the 83 drugs identified, they deeply studied Niclosamide, an approved antihelminthic drug, which exert also inhibitory effects against virus replication [165]. The authors found that Niclosamide suppresses the activity of TMEM16F/Anoctamin6, a calcium-activated ion channel that mediates phosphatidylserine exposure on the cell surface [165, 166]. Interestingly, the results of a phase I trial of a formulation of niclosamide as a potent anti-SARS-CoV-2 agent have been recently published [167].

### 5.2. Targeting Cellular Proteases

Since the S protein priming of CoVs by cellular proteases is necessary for receptor binding and membrane fusion, these proteases can serve as targets (Table 2) for developing inhibitors of S protein-mediated viral entry into the target cells [49].

Coronaviruses enter cells via two distinct pathways, one mediated by TMPRSS2 at the cell surface and the other mediated by cathepsins in the endosome [22, 23, 25]. Since endosomal cathepsins (B/L) and TMPRSS2 can activate CoV S-mediated virus-cell entry and uptake, treatment of cells with either cathepsin (B/L) inhibitors (i.e., MDL28170 or teicoplanin) or TMPRSS2 inhibitors (i.e., camostat mesylate or peptidic inhibitors) can block virus entry into target cells [25, 49, 81, 145, 168]. SARS-CoV infection was blocked by specific inhibitors of the pH-sensitive endosomal protease cathepsin L. S-mediated entry into Vero E6 and 293T/hACE2 cells was blocked by leupeptin, Z-lll-FMK (an inhibitor of both CTSB and CTSL), and E64c (an inhibitor of cysteine proteases) [21].

Further studies showed that inhibitors of cathepsin L blocked infection by both SARS-CoV and pseudotype expressing the SARS-CoV S protein, while not affecting the infection by either HCoV-NL63 or a retrovirus pseudotyped with the HCoV-NL63 S protein in HEK293T cells expressing Cathepsins and ACE2. In addition, expression of exogenous cathepsin L substantially enhanced infection mediated by the SARS-CoV S protein and by filovirus GP proteins but not by the HCoV-NL63 S protein or the vesicular stomatitis virus G protein [124].

The role of cellular proteases in virus entry into cells was observed also for MERS-CoV, as the protease inhibitors camostat and MDL28170 efficiently inhibited entry of pseudotyped MERS-CoV into the human colorectal cell line Caco-2 cells [81]. In fact, simultaneous treatment with inhibitors of cathepsin L and TMPRSS2 completely blocked virus entry into Vero-TMPRSS2 cells, indicating that MERS-CoV employs both the cell surface and the endosomal pathway to infect TMPRSS2-expressing Vero cells. In contrast, a single camostat treatment suppressed MERS-CoV entry into human bronchial submucosal gland-derived Calu-3 cells by 10-fold and virus growth by 270-fold, although treatment with both camostat and (23,25)-trans-epoxysuccinyl-L-leucylamindo-3-methylbutane ethyl ester (EST), a cathepsin inhibitor, or treatment with leupeptin, an inhibitor of cysteine, serine, and threonine peptidases, was no more efficacious than treatment with camostat alone [25].

Recently, Hoffmann et al. showed that SARS-CoV-2-S-driven entry was fully inhibited when both camostat mesylate and E-64d, an inhibitor of cathepsin B/L, were added to Caco-2, TMPRSS2-expressing Vero, and 293T (transiently expressing ACE2) cell cultures, indicating that SARS-CoV-2 S protein can use both cathepsin B/L as well as TMPRSS2 for priming in these cell lines. Further, camostat mesylate significantly reduced MERS-CoV-S-, SARS-CoV-S-, and SARS-CoV-2-S-driven and authentic SARS-CoV-2 entry into the Calu-3 cells. Similarly, camostat mesylate treatment inhibited SARS-S- and SARS-2-S- but not VSV-G-driven entry into primary human lung cells, suggesting that SARS-CoV-2 can use TMPRSS2 for S protein priming and camostat mesylate blocks SARS-CoV-2 infection of lung cells [131].

In another study, camostat partially blocked infection by SARS-CoV and HCoV-NL63 in HeLa cells expressing ACE2 and TMPRSS2. Moreover, simultaneous treatment with camostat and EST efficiently prevented both cell entry and the multistep growth of SARS-CoV in human Calu-3 cells [105]. These observations suggest that inhibition of both proteases is required for viral entry inhibition [22, 25, 105, 169].

It has also been shown by Bergeron and colleagues that furin, a ubiquitously expressed protease, plays a key role in protease-activated CoV S-based fusion. The convertase inhibitor, membrane-permeable peptide decanoyl-RVKR-chloromethylketone (dec-RVKR-cmk) significantly reduced pro-S cleavage, viral entry, and infection. In addition, inhibition of processing by dec-RVKR-cmk completely abrogated the virus-induced cellular cytopathicity [148]. Moreover, siRNA silencing of furin activity decreased MERS-CoV S-mediated entry, and blockage of furin cleavage at the S cleavage sites significantly reduces virus infection [99]. However, this protease appears to be indispensable, and blockade of it does not affect S protein-driven cell-cell and virus-cell fusion of MERS-CoV, although it can reduce the processing of S protein in infected cells.

Finally, furin inhibition by decanoyl-RVKR-chloromethylketone (CMK) and naphthofluorescein has been found to inhibit syncytia formation and decrease SARS-CoV-2-induced cytopathic effects and viral production [170]. In particular, it has been reported that CMK blocks virus entry while naphthofluorescein suppresses mainly viral RNA transcription, thus, further suggesting that furin inhibitors may be promising antiviral agents for SARS-CoV-2 infection.

## 6. Conclusions

CoVs entry into cells is based on (1) binding of viral S protein to a specific receptor and (2) priming it with host cellular proteases such as TMPRSS2, furin, and cathepsin L. Different strategies are under investigation for inhibiting the binding of the S protein to the cellular receptors or the fusion process using antireceptor antibodies, soluble receptors, anti-S1, or -S2 antibodies or targeting cellular proteases. These strategies may offer opportunities to identify new antiviral drugs and are of particular relevance for vaccine development against highly pathogenic CoVs and particularly new variants of SARS-CoV-2. Inhibitors of viral entry appear to be a promising therapeutic strategy due to their ability to block the very first step of the viral life cycle and less toxic potentials because the membrane permeability may not be necessary. In this regard, identification of relevant in vitro models and cell lines permissive for CoVs facilitates further laboratory investigations; however, these cellular models have limitations, due to their inability to recapitulate complex and dynamic responses or cell-cell interactions that naturally occur in the organism.

Primary culture systems such as HBEpC, HAE culture models, human-derived organoids, or pseudostratified epithelia may be promising models to study CoVs. In particular, the HAE culture model expressing relevant receptors for CoVs can recapitulate not only the morphological features of the human upper airway, allowing visualization of the high levels of replication and destructive nature of viruses, but also provides an excellent model for studying viral entry, and comparing the responses of the lung to newly emerged viruses, including SARS-CoV-2 infections. Finally, organoids which are miniaturized, simplified, and three-dimensional versions of an organ produced in vitro can be a valid model to study tropisms and complex physiological or pathological processes of highly pathogenic CoVs, similarly to the in vivo situation.

## Figures and Tables

**Table 1:** CD26/DPP4 and ACE2 receptor expression in different primate cell lines.

Cell line	Origin	Expression marker	Level	Method of evaluation	Virus	Reference
Nonhuman cells:						
Vero, Vero E6	African green monkey kidney	CD26/DPP4	N/R	Immunofluorescence	MERS-CoV	[90]
		CD26/DPP4	High	Flow cytometry (FACS), immunofluorescence	MERS-CoV	[27]
		CD26/DPP4	High	Flow cytometry (FACS), western blot	MERS-CoV	[28]
		CD26/DPP4	High	Flow cytometry (FACS), protein electrophoresis	MERS-CoV	[29]
		CD26/DPP4	Low	Real-time RT-PCR, western blot	MERS-CoV	[25]
		CD26/DPP4	Int.	Western blot	MERS-CoV	[79]
		CD26/DPP4	High	Flow cytometry (FACS)	MERS-CoV	[83]
		CD26/DPP4	Int.	Quantitative RT-PCR	MERS-CoV	[91]
		CD26/DPP4	Int.	Quantitative RT-PCR, flow cytometry (FACS)	MERS-CoV	[92]
		CD26/DPP4	High	Immunofluorescence	MERS -CoV	[93]
		ACE2	High	Real-time RT-PCR	SARS-CoV	[17]
		ACE2	High	RT-PCR	SARS-CoV	[60]
		ACE2	Int.	Immunofluorescence	SARS-CoV	[93]
		ACE2	High	Western blot	SARS-CoV	[94]
		ACE2	Int.	Flow cytometry (FACS)	SARS-CoV	[95]
		ACE2	High	Immunofluorescence	SARS-CoV	[96]

LLC-MK2	Rhesus monkey kidney	ACE2	High	Flow cytometry (FACS), immunofluorescence	SARS-CoV	[97]
		ACE2	High	Immunostaining	HCoV-NL63	[98]

Human cells:						
MRC-5	Lung cells Medical Research Council-5	CD26/DPP4	High	Quantitative RT-PCR	MERS -CoV	[99]

A549	Human lung adenocarcinoma	CD26/DPP4	Low	Enzymatic activities, immunohistochemistry (IHC)	-	[100]
		CD26/DPP4	N/R	Flow cytometry (FACS)	-	[101]
		CD26/DPP4	N/R	Immunofluorescence	MERS -CoV	[90]
		CD26/DPP4	Low	Western blot	MERS-CoV	[79]
		ACE2	N/R	Western blot	-	[102]
		ACE2	High	Immunostaining	SARS-CoV	[58]
		ACE2	Low	Immunoblot	SARS-CoV	[70]

Calu-3	Human lung cancer cells	CD26/DPP4	High	Immunofluorescence	MERS-CoV	[30]
		CD26/DPP4	High	Real-time RT-PCR, western blot	MERS-CoV	[25]
		CD26/DPP4	Int.	Real-time RT-PCR	MERS-CoV	[103]
		CD26/DPP4	High	Flow cytometry (FACS)	MERS-CoV	[82]
		ACE2	High	Flow cytometry (FACS), immunofluorescence	SARS-CoV	[104]
		ACE2	High	Real-time RT-PCR	SARS-CoV	[105]
		ACE2	High	Flow cytometry (FACS), western blot	-	[106]
		ACE2	Low	Immunofluorescence	SARS-CoV	[96]

HEp-2	Human respiratory tract	ACE2	High	RT-PCR	SARS-CoV	[60]
		CD26/DPP4	Neg.	Western blot	MERS-CoV	[79]

Huh-7	Hepatocellular carcinoma	CD26/DPP4	High	Flow cytometry (FACS), immunofluorescence	MERS-CoV	[27]
		CD26/DPP4	High	Flow cytometry (FACS), western blot	MERS-CoV	[28]
		CD26/DPP4	High	Flow cytometry (FACS), protein electrophoresis	MERS-CoV	[29]
		CD26/DPP4	N/R	Flow cytometry (FACS)	MERS-CoV	[107]
		CD26/DPP4	High	Real-time RT-PCR	MERS-CoV	[103]
		CD26/DPP4	High	Western blot	MERS-CoV	[79]
		CD26/DPP4	Int.	Quantitative RT-PCR	MERS-CoV	[99]
		ACE2	High	Real-time RT-PCR	SARS-CoV	[17]
		ACE2	High	RT-PCR	SARS-CoV	[60]
		ACE2	Neg.	Flow cytometry (FACS), western blot	-	[106]

LoVo	Human colorectal adenocarcinoma	ACE2	Int.	Real-time RT-PCR	SARS-CoV	[61]
		CD26/DPP4	High	Real-time RT-PCR	MERS-CoV	[103]

Caco-2	Human intestinal tract	CD26/DPP4	Int.	Western blot	MERS-CoV	[79]
		CD26/DPP4	High	Quantitative RT-PCR	MERS-CoV	[91]
		CD26/DPP4	High	Quantitative RT-PCR, flow cytometry (FACS)	MERS-CoV	[92]
		ACE2	Low	Flow cytometry (FACS), western blot	-	[106]
		ACE2	High	Immunofluorescence	SARS-CoV	[96]
		ACE2	High	RT-PCR	-	[108]

Abbreviations: ACE2: angiotensin-converting enzyme 2; DPP4: dipeptidyl peptidase 4; CD26: cluster of differentiation 26; SARS-CoV: severe acute respiratory syndrome coronavirus; MERS-COV: Middle East respiratory syndrome coronavirus; Int.: intermediate; N/R: not reported.

**Table 2:** In vitro models for studying cell entry and tissue tropism of different pathogenic coronaviruses.

Cell lines	Origin	Virus	Main finding related to cell entry and tissue tropism	Reference
Nonhuman cells:				
Vero	African green monkey kidney	SARS-CoV	TMPRSS2 is involved in virus entry.	[22]
		SARS-CoV	Cathepsin L inhibitor blocked SARS-CoV infection.	[124]
		SARS-CoV	Small-molecule compounds could perturb the infectivity of the virus.	[125]
		SARS-CoV	Sensitivity of S-mediated entry to protease inhibitors.	[21]
		SARS-CoV	ACE2 as SARS-CoV S pseudovirion receptor for entry.	[8]
		SARS-CoV	Receptor blocked by polyclonal goat anti-hACE2.	[97]
		SARS-CoV-2	ACE2 as SARS-CoV-2 S pseudovirion receptor for entry.	[8]
		MERS-CoV	Furin inhibitor significantly decreased S-mediated entry.	[99]
		MERS-CoV	DPP4 as the receptor for viral entry.	[28, 29]
		MERS-CoV	DPP4 is expressed on permissive cell.	[90]
		MERS-CoV	HR2P significantly inhibited virus replication.	[46]
		MERS-CoV	Simultaneous treatment with inhibitors of cathepsin L and TMPRSS2 completely blocked virus entry.	[25]

VeroE6	African green monkey kidney	SARS-CoV	Rabbit antisera effectively blocked binding of S1 to ACE2.	[126]
		SARS-CoV	ACE2 as the receptor for viral entry.	[127]
		SARS-CoV	Anti-ACE2 blocked viral entry.	[31]
		SARS-CoV	Protease Inhibitors (leupeptin, Z-lll-FMK) blocked SARS-CoV S-mediated entry.	[21]
		SARS-CoV	Proteases enhanced virus entry.	[128]
		SARS-CoV	Vimentin as a coreceptor involved in the virus entry.	[68]
		SARS-CoV	Different host cell proteases activate SARS-S for virus–cell and cell–cell fusion.	[129]
		SARS-CoV	Cholesterol extraction by M*β*CD treatment could reduce the expression level of cell surface ACE2 at a dose-dependent manner.	[95]
		SARS-CoV-2	SARS-CoV S polyclonal Abs inhibited SARS-CoV-2 spike mediated entry.	[39]

LLC-MK2	Rhesus monkey kidney	SARS-CoV	ACE2 as the receptor for viral entry.	[97]

COS-7	Monkey kidney	MERS-CoV	Expression of human and bat DPP4 allowed MERS-CoV S1–Fc cell surface binding, and viral entry.	[29]

BHK	Baby hamster kidney	SARS-CoV	ACE2 as the receptor for viral entry.	[97]
		SARS-CoV	Proteolytic cleavage within S2 exposes a novel internal fusion peptide for SARS-CoV S.	[130]
		SARS-CoV, SARS-CoV-2	ACE2 as the receptor for viral entry.	[131]
		SARS-CoV-2	ACE2 and host proteases requirement for viral entry.	[132]
		MERS-CoV	hDPP4 transfected cells became permissive.	[133]
		MERS-CoV	Virus could infect human and bat cells expressing DPP4.	[134]
		MERS-CoV	Specific binding between CD26 and MERS-CoV RBD.	[48]

C6	Rat glioma-derived	SARS-CoV	No apparent cytopathic effects (CPE) by infection but produced virus with infectivity of 102–5 per ml. No expression of ACE2	[117]

Human cells:				
A549	Lung adenocarcinoma	MERS-CoV	DPP4 is expressed on permissive cells.	[90]

OL	Oligodendroglioma-derived	SARS-CoV	No apparent cytopathic effects (CPE) by infection but produced virus with infectivity of 102–5 per ml. No expression of ACE2	[117]

Calu-3	Airway epithelium	SARS-CoV	hACE2 was required for cell entry.	[118]
		SARS-CoV	Anti-ACE2 Ab blocked the cell entry in a dose-dependent manner.	[104]
		SARS-CoV	ACE2, the SARS-CoV S pseudovirion receptor for entry.	[8]
		SARS-CoV	Simultaneous treatment of the cells with camostat and EST efficiently prevented both cell entry and the multistep growth of the virus in the cells.	[105]
		SARS-CoV-2	ACE2 as the SARS-CoV-2 S pseudovirion receptor for entry.	[8]
		MERS-CoV	DPP4 is expressed on permissive cells.	[30]
		MERS-CoV	TMPRSS2 inhibitor (camostat) blocked virus entry.	[25]

Huh-7	Hepatocellular carcinoma	SARS-CoV	S-mediated entry of pseudotypes requires low pH, S is a target for neutralizing antibodies.	[135]
		SARS-CoV	ACE2 as the SARS-CoV S pseudovirion receptor for entry.	[8]
		SARS-CoV-2	ACE2 as the SARS-CoV-2 S pseudovirion receptor for entry.	[8]
		MERS-CoV	Anti-CD26 mAbs (2F9) inhibited viral entry.	[78]
		MERS-CoV	pAbs to the MERS-CoV S1 efficiently neutralize virus infection.	[136]
		MERS-CoV	Furin inhibitor significantly decreased S-mediated entry.	[99]
		MERS-CoV	DPP4 as the receptor for viral entry.	[28, 29]
		MERS-CoV	Nanobodies significantly blocked RBD binding to DPP4.	[137]
		MERS-CoV	Antihuman CD26/DPP4 antibody inhibited MERS-CoV infection.	[27]
		MERS-CoV	S protein-mediated cell–cell fusion and syncytium formation.	[46]
		MERS-CoV	Interaction between recombinant RBDs and DPP4.	[107]

HEK293T	Embryonic kidneys	SARS-CoV	Cathepsin L inhibitor blocked S mediated pseudovirus entry in ACE2+ cells.	[124]
		SARS-CoV	Lactoferrin blocked the binding of S protein to ACE2 transfected cells.	[138]
		SARS-CoV-2	Binding of polyclonal rabbit anti-SARS S1 antibodies to SARS-CoV-2.	[8]
		MERS-CoV	hDPP4 transfected cells became permissive.	[133]
		MERS-CoV	High levels of hDPP4 and furin enhanced viral entry. Knocked down furin expression with siRNA significantly reduced the pseudovirus entry.	[10]

293T	Embryonic kidney epithelial	SARS-CoV	S Protein efficiently binds ACE2.	[139]
		SARS-CoV	Neutralization of SARS pseudovirus infection by mouse antisera.	[140]
		SARS-CoV	TMPRSS2 protease-dependent viral entry.	[141]
		SARS-CoV	Protease inhibitors (leupeptin, E64c) blocked SARS-CoV S-mediated entry.	[21]
		SARS-CoV	Cytoplasmic domain was not essential for ACE2-mediated viral entry; soluble ACE2 inhibited S-bearing pseudotype entry.	[60]
		SARS-CoV	Rabbit antisera effectively blocked binding of S1 to ACE2.	[126]
		SARS-CoV	ACE2 was required for entry.	[31]
		SARS-CoV	SARS-CoV RBD protein inhibited virus entry.	[18]
		SARS-CoV	ACE2 as the SARS-CoV S pseudovirion receptor for entry.	[8]
		SARS-CoV	MAbs inhibited RBD-Fc binding to ACE2.	[142, 143]
		SARS-CoV	A compound (designated VE607) inhibited pseudovirus entry.	[125]
		SARS-CoV	ACE2 as the SARS-CoV S pseudovirion receptor for entry.	[81]
		SARS-CoV	TMPRSS2-mediated proteolysis of both S and ACE2 enhanced viral entry.	[23]
		SARS-CoV	S-mediated entry of lentiviral-based vectors.	[144]
		SARS-CoV	Proteases activated SARS-S-driven virus-cell fusion.	[145]
		SARS-CoV	Host cell proteases activated SARS-S for virus–cell and cell–cell fusion.	[129]
		SARS-CoV-2	ACE2 as the SARS-CoV-2 S pseudovirion receptor for entry.	[8]
		SARS-CoV-2	Protease was required for S-driven entry.	[131]
		SARS-CoV-2	SARS-CoV-2 RBD protein inhibited both SARS- and SARS-CoV-2 entry.	[18]
		MERS-CoV	MERS-CoV RBD inhibited MERS-CoV pseudovirus entry.	[18]

HeLa	Cervical adenocarcinoma	SARS-CoV	TMPRSS2 enhanced pseudotyped SARS-S and authentic SARS-CoV entry, and camostat blocked it.	[105]
		SARS-CoV-2	ACE2 was required for viral entry.	[146]

JKT-hCD26	Human T cell leukemia	MERS-CoV	Anti-CD26 mAbs (2F9) inhibited viral entry.	[78]

LoVo	Colorectal adenocarcinoma	SARS-CoV	ACE2 is expressed on permissive cell.	[61]
		MERS-CoV	DPP4 is expressed on permissive cell.	[30]

Caco-2	Colorectal adenocarcinoma	SARS-CoV-2	Protease was required for S-driven entry.	[131]
		MERS-CoV	Protease (TMPRSS2 and cathepsin B/L) could activate EMC-S for entry.	[81]

Primary culture				
HBEpC	Primary bronchial epithelial cell	SARS-CoV, MERS-CoV	Specific receptors were needed for cell entry.	[93]

HREpC	Primary renal epithelial cells	SARS-CoV, MERS-CoV	Specific receptors were needed for cell entry.	[93]

HAE	Airway epithelium	SARS-CoV	hACE2 as the primary receptor for entry.	[118]
		SARS-CoV	S protein-pseudotyped FIV infected differentiated cells abundantly express ACE2 from the apical surface.	[70]
		MERS-CoV	Both type I and type III IFN efficiently reduced MERS-CoV replication.	[147]
		MERS-CoV	TMPRSS2 inhibitor (camostat) blocked virus entry.	[25]

NHBE	Normal human bronchial epithelial	MERS-CoV	Furin inhibitor significantly decreased S-mediated entry.	[99]

3D human organoids	Induced pluripotent stem cells- (iPSCs-) derived	SARS-CoV-2	The impact of SARS-CoV-2 as a neurotropic virus and emphasize that brain organoids could model CNS pathologies of COVID-19	[110]
		SARS-CoV-2	Neuronal infection can be prevented either by blocking ACE2 with antibodies or by administering cerebrospinal fluid from a COVID-19 patient.	[113]
		SARS-CoV-2	Using hPSCs to generate multiple different cell and organoid derivatives to study the viral tropism and cellular responses to infection.	[112]
		SARS-CoV-2	SARS-CoV-2 can infect neural cells. detected the expression of the ACE2 receptor, but not TMPRSS2, in the model.	[111]
		SARS-CoV-2	ACE2 expresses in cultured human pluripotent stem cell- (PSC-) derived mixed neurons.	[114]
